# Association between the ratio of serum creatinine to cystatin C and bone mineral density in Chinese older adults patients with type 2 diabetes mellitus

**DOI:** 10.3389/fnut.2022.1035853

**Published:** 2022-10-21

**Authors:** Ting Gao, Fupeng Liu, Bo Ban, Yue Hou, Guangxin Li, Mingming Jiang, Qing Yang, Mei Zhang

**Affiliations:** ^1^Department of Clinical Medicine, Jining Medical University, Jining, Shandong, China; ^2^Department of Endocrinology, Affiliated Hospital of Jining Medical University, Jining, Shandong, China; ^3^Department of Nutrition, Affiliated Hospital of Jining Medical University, Jining, Shandong, China

**Keywords:** type 2 diabetes, creatinine, cystatin C, Cre/CysC, bone mineral density

## Abstract

**Background:**

The ratio of creatinine to cystatin C (Cre/CysC), a marker of muscle function and muscle mass, can be used to predict sarcopenia in different populations. Since sarcopenia is closely associated with osteoporosis, this study investigated the association between Cre/CysC and bone mineral density (BMD) in patients with type 2 diabetes mellitus (T2DM).

**Method:**

This cross-sectional study included 391 Chinese patients with T2DM. General information, biochemical indicators, and the BMD of lumbar spine (LS), femoral neck (FN), and total hip (TH) were measured.

**Results:**

Pearson correlation analysis showed that Cre/CysC was significantly positively correlated with the BMD of LS (*r* = 0.170, *p* = 0.001), FN (*r* = 0.178, *p* < 0.001), and TH (*r* = 0.205, *p* < 0.001). The results of stepwise linear regression suggested that Cre/CysC was the only biochemical predictor of the BMD at three sites (LS: β = 0.137, *p* = 0.01; FN: β = 0.097, *p* = 0.038; TH: β = 0.145, *p* = 0.002).

**Conclusion:**

In older patients with T2DM, high Cre/CysC value is independently positively associated with BMD and hence, Cre/CysC may serve as a valuable marker of osteoporosis.

## Introduction

In the past few years, the incidence of diabetes has increased significantly. According to the 2021 International Diabetes Federation report, diabetes affects approximately 537 million adults aged 20–79 years worldwide, and it is predicted that 783 million people will have diabetes by 2045 (1); thus, diabetes has become an increasingly serious global health problem. Some studies have documented that the risk of osteoporosis is significantly higher in patients with type 2 diabetes mellitus (T2DM) than in those without T2DM (2). The guidelines for primary osteoporosis diagnosis mention bone mineral density (BMD) as an essential marker for the diagnosis of osteoporosis (3). A recent large-scale study assessing the determinants of clinical risk of fractures by genome-wide analysis confirmed that only BMD had a major effect on fractures, and low BMD was a pivotal cause of osteoporotic fracture (4). Therefore, in such patients, early identification of risk factors related to BMD is crucial for the early diagnosis and treatment of osteoporosis and the prevention of adverse fracture outcomes.

Sarcopenia is characterized by both decreased muscle mass and function (5). Compared with the population without diabetes, the incidence of sarcopenia in patients with T2DM increased significantly (6). Studies have shown that muscle mass and muscle function are closely related to bone health, which mainly manifested in the increased risk of falls and fractures (7–9). In the T2DM population, the presence of risk factors, such as reduced physical activity, obesity, and steroid hormone use, will further aggravate muscle and bone diseases.

Creatinine is the product of normal catabolism of muscle tissue and is proportional to muscle mass; however, as renal function affects creatinine level, it cannot be used as a potential indicator of muscle mass in clinical settings. Cystatin C, an endogenous protein, can reliably reflect the glomerular filtration rate (10). Therefore, the ratio of creatinine to cystatin C (Cre/CysC) is considered to be the ratio of muscle mass to total body mass after adjusting for renal function, which reflects muscle volume and is closely related to skeletal muscle mass volume (11). Based on this theory, previous studies have confirmed that Cre/CysC can accurately predict the incidence of sarcopenia in different populations. Osaka et al. have indicated that decreased Cre/CysC is considered an independent predictor of sarcopenia in patients with T2DM (12). Considering the close relationship between muscle mass, muscle function, and osteoporosis, Cre/CysC may be a reliable index for predicting osteoporosis. A study report on indicators of bone properties in older adults showed that Cre/CysC might be used as a biochemical marker of bone property independent of muscle mass in the population with preserved renal function (13). In another study on Japanese postmenopausal women, Cre/CysC had a positive correlation with BMD, and was suggested to be one of the surrogate marker candidates of osteoporosis (3). However, to date, the relationship between Cre/CysC and BMD in patients with T2DM has not been researched. Given these backgrounds, this study aimed to clarify the relationship between serum Cre/CysC and BMD in older patients with T2DM.

## Materials and methods

### Study patients

This study analyzed data collected from 391 patients at the Department of Endocrinology, Affiliated Hospital of Jining Medical College between June 2021 and April 2022. Information related to demographics, health status and function, health outcomes, including blood biomarker measurements, and BMD measurements, were collected. All patients had been diagnosed with T2DM at baseline according to the 2019 World Health Organization (WHO) standards. The exclusion criteria were as follows: patients younger than 50 years and premenopausal women; patients without anthropometric measures; those lacking blood biochemical index data of creatinine and cystatin C; those lacking BMD examination; those with the presence of malignant tumor, serious liver, kidney, heart disease, or metabolic diseases, such as those involving the pituitary, thyroid, and adrenal glands; and those receiving hemodialysis, immunosuppressive drugs, or drugs that affect bone metabolism, including vitamin D and calcium. The Human Ethics Committee of the Affiliated Hospital of Jining Medical University approved this study.

### Body composition measurement

Information on the clinical characteristic, including sex, age, weight, height, body mass index (BMI), blood pressure, duration of diabetes, disease history (such as hypertension, hyperlipidemia, cerebrovascular accident, coronary artery disease, and kidney disease), and drug consumption history were obtained from the electronic medical records of the hospital. Height and weight were measured accurately to 0.1 cm and 0.1 kg, respectively. BMI was calculated as weight (kilograms)/height (meters squared). The duration of diabetes was calculated from the time when T2DM was diagnosed based on the patient’s medical records to the year when blood and BMD tests were performed.

### Laboratory measurements

After fasting for 8–12 h, fasting blood samples were obtained from all patients for laboratory analyses. Glucose metabolism indices included the following: glycated hemoglobin (HbA1c), fasting blood glucose (FBG), and serum C-peptide; renal function indices: uric acid (UA), creatinine, and cystatin C; liver function indices: total protein, albumin (ALB), total bilirubin, direct bilirubin, and indirect bilirubin; serum lipid metabolism indices: total cholesterol, triglycerides (TG), high-density lipoprotein (HDL), low-density lipoprotein, and very low-density lipoprotein; thyroid function indices: free triiodothyronine, free thyroxine, and thyroid-stimulating hormone. Parathyroid hormone, calcitonin, 25-hydroxy-vitamin D_3_, serum calcium, serum magnesium, and serum phosphorus were also measured. In particular, creatinine (mg/dl) was determined using a sarcosine oxidase method (Diacegene, Sichuan, China), and cystatin C was measured by the particle-enhanced turbidimetric assay (Zybio, Chongqing, China). Serum creatinine (mg/L) was divided by serum cystatin C to calculate Cre/CysC (mg/L).

### Bone mineral density measurement

Dual-energy X-ray absorptiometry (DEXA) was used to measure BMD (grams per square centimeter). Each patient was measured at three sites: the lumbar spine (LS), femoral neck (FN), and total hip (TH). BMD was measured according to the WHO standards as follows: normal, *T*-scores ≥−1.0; osteopenia, *T*-scores between −1.0 and −2.5; and osteoporosis, *T*-scores <−2.5 (14).

### Statistical analysis

All statistical analyses were performed using the SPSS software (V.26.0). Continuous data with a normal distribution are expressed as mean values ± SD, whereas non-normal distributed data are expressed as medians (quartile). An independent sample *t*-test or Mann–Whitney U test was used to compare the data between the two groups. Categorical data were presented in terms of frequency or percentage and analyzed with Chi-square tests. Pearson’s correlation coefficients were used to examine the correlation between biochemical indices and BMD. Variables that significantly correlated with BMD were included in the multiple stepwise linear regression analysis, and multiple stepwise linear regression analysis was performed using the stepwise selection of the variables to determine the variables independently related to the BMD of the LS, FN, and TH. Receiver operating characteristic (ROC) curves were plotted with osteopenia and osteoporosis as state variable. The area under the curve (AUC) and Youden index were calculated to get cut-off points of Cre/CysC. All tests for statistical significance were two-tailed, and statistical significance was set at *p* < 0.05.

## Results

### Patient characteristics

The clinical and laboratory characteristics of the patients are summarized in Table 1. Among the 391 patients, 202 were men and 189 were women. The average age of patients was 61.17 ± 9.47 years. Patients had an average duration of 10 years of having T2DM. The mean HbA1c level was 8.83 ± 2.06%, which was significantly higher in women (9.07 ± 2.07) than in men (8.61 ± 2.04). The mean creatinine level was 7.03 ± 2.52 mg/L, which was significantly higher in men (7.74 ± 2.35) than in women (6.27 ± 2.38). The mean cystatin C level was 1.11 ± 0.40 mg/L, which was higher in women (1.12 ± 0.50) than in men (1.10 ± 0.27), although the difference was not statistically significant. The average Cre/CysC value was 6.40 ± 1.52, which was significantly higher in men (7.04 ± 1.46) than in women (5.72 ± 1.26). The average BMD of LS, FN, and TH were 1.07 ± 0.19, 0.89 ± 0.16, and 0.94 ± 0.16, respectively. The BMD of men at the three sites was significantly higher than that of women (Table 1).

**TABLE 1:** Characteristics of patients included in this study.

Characteristic	Total (*n* = 391)	Male (*n* = 202)	Female (*n* = 189)	*P*-value
AGE (years)	61.17 ± 7.47	60.14 ± 7.03	62.28 ± 7.79	0.005
DURATION (years)	10.00 (5.00, 17.00)	10.00 (5.00, 17.00)	10.00 (4.25, 16.00)	0.47
BMI (kg/m^2^)	25.51 ± 3.89	25.47 ± 3.81	25.55 ± 3.99	0.838
HBA1C (%)	8.83 ± 2.06	8.61 ± 2.04	9.07 ± 2.07	0.029
CREATININE (mg/L)	7.03 ± 2.52	7.74 ± 2.35	6.27 ± 2.38	<0.001
CYSTATIN C (mg/L)	1.11 ± 0.40	1.10 ± 0.27	1.12 ± 0.50	0.686
CRE/CYSC	6.40 ± 1.52	7.04 ± 1.46	5.72 ± 1.26	<0.001
UA (μmol/L)	282.98 ± 93.83	302.82 ± 93.10	261.67 ± 90.10	<0.001
TP (g/L)	65.30 ± 6.30	65.63 ± 6.42	64.95 ± 6.17	0.284
ALB (g/L)	41.43 ± 4.45	42.24 ± 4.03	40.58 ± 4.72	<0.001
TBIL (μmol/L)	14.38 ± 5.33	15.42 ± 5.76	13.27 ± 4.60	<0.001
DBIL (μmol/L)	3.64 ± 1.63	3.72 ± 1.69	3.55 ± 1.57	0.288
IDBIL (μmol/L)	10.80 ± 4.37	11.69 ± 4.73	9.85 ± 3.74	<0.001
TG (mmol/L)	1.25 (0.90, 1.89)	1.28 (0.86, 2.03)	1.23 (0.90, 1.74)	0.368
TCH (mmol/L)	4.37 ± 1.22	4.22 ± 1.28	4.52 ± 1.14	0.017
HDL (mmol/L)	1.24 ± 0.29	1.20 ± 0.28	1.29 ± 0.29	0.001
LDL (mmol/L)	2.61 ± 0.85	2.50 ± 0.85	2.71 ± 0.83	0.015
VLDL (mmol/L)	0.43 (0.31, 0.57)	0.41 (0.30, 0.59)	0.44 (0.33, 0.56)	0.591
FBG (mmol/L)	7.83 ± 3.52	7.75 ± 3.72	7.91 ± 3.31	0.659
FCP (ng/mL)	1.93 ± 1.23	1.86 ± 1.15	2.00 ± 1.31	0.297
FT3 (pmol/L)	4.55 ± 1.80	4.60 ± 0.68	4.49 ± 2.51	0.554
FT4 (pmol/L)	16.97 ± 4.34	17.13 ± 2.38	16.79 ± 5.76	0.443
TSH (miu/L)	2.18 ± 1.76	1.96 ± 1.56	2.43 ± 1.93	0.01
PTH (pg/mL)	35.08 ± 16.52	34.00 ± 15.70	36.23 ± 17.34	0.205
CT (pg/mL)	5.48 (3.29, 12.03)	9.03 (4.48, 13.42)	3.71 (2.54, 5.70)	<0.001
25-(OH)D_3_ (ng/mL)	17.48 ± 7.28	18.98 ± 7.66	15.83 ± 6.48	<0.001
CA (mmol/L)	2.27 ± 0.12	2.27 ± 0.12	2.26 ± 0.11	0.395
P (mmol/L)	1.24 ± 0.20	1.22 ± 0.19	1.27 ± 0.21	0.019
MG (mmol/L)	0.91 ± 0.10	0.92 ± 0.08	0.90 ± 0.12	0.091
LS BMD (G/CM^2^)	1.07 ± 0.19	1.11 ± 0.17	1.02 ± 0.19	<0.001
FN BMD (G/CM^2^)	0.89 ± 0.16	0.95 ± 0.15	0.83 ± 0.15	<0.001
TH BMD (G/CM^2^)	0.94 ± 0.16	1.00 ± 0.15	0.89 ± 0.15	<0.001

BMI, body mass index; HbA1c, hemoglobin A1c; Cre/CysC, creatinine to cystatin C ratio; UA, uric acid; TP, total protein; ALB, albumin; TBIL, total bilirubin; DBIL, direct bilirubin; IDBIL, indirect bilirubin; TG, triglycerides; TCH, total cholesterol; HDL, high-density lipoproteins; LDL, low-density lipoproteins; VLDL, very low-density lipoprotein; FBG, fasting blood glucose; FCP, fasting C-peptide; FT3, free triiodothyronine; FT4, free thyroxine; TSH, thyroid-stimulating hormone; PTH, parathyroid hormone; CT, calcitonin; 25-(OH)D_3_, 25-hydroxy-vitamin D_3_; Ca, calcium; P, phosphorus; Mg, magnesium; LS BMD, lumbar spine bone mineral density; FN BMD, femoral neck bone mineral density; TH BMD, total hip bone mineral density.

### Correlations between clinical factors and bone mineral densities

**FIGURE 1:**
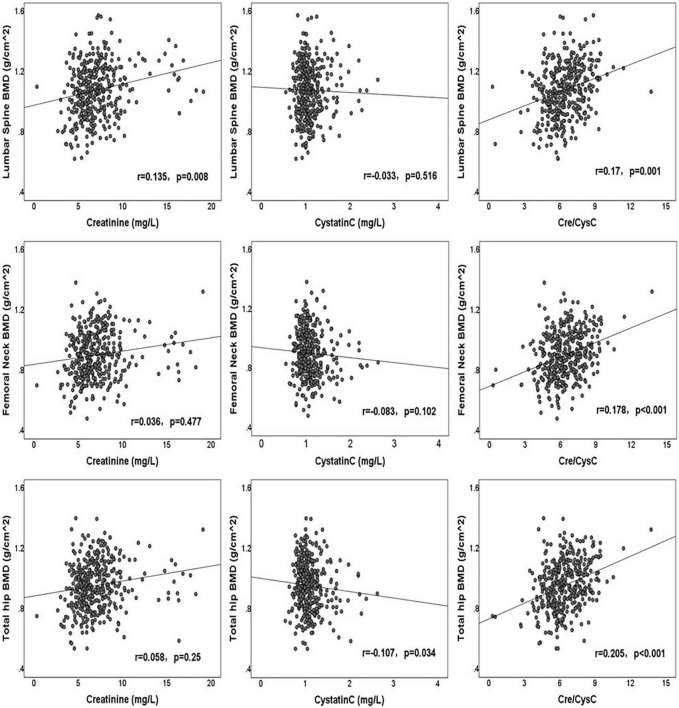
Scatter plot of creatinine, cystatin C, creatinine/cystatin C, and BMDs.

The correlations between clinical factors and BMDs of the LS, FN, and TH are shown in Table 2. Creatinine had a significantly positive correlation with LS BMD (*r* = 0.135, *p* = 0.008), although without significant correlation with FN BMD (*r* = 0.036, *p* = 0.477) and TH BMD (*r* = 0.058, *p* = 0.250). Cystatin C was significantly negatively correlated with the BMD of TH (*r* = −0.107, *p* = 0.034), but was not associated with the BMD of LS (*r* = −0.033, *p* = 0.516) and FN (*r* = −0.083, *p* = 0.102). Cre/CysC had a strong positive relationship with the BMD of LS (*r* = 0.170, *p* = 0.001), FN (*r* = 0.178, *p* < 0.001), and TH (*r* = 0.205, *p* < 0.001). The correlations between creatinine, cystatin C, Cre/CysC, and BMD are shown in Figure 1. In addition, we observed significant correlations between the age, BMI, HbA1c, UA level, HDL, serum phosphorus level, and the BMDs of LS, FN, and TH. The duration of diabetes was only related to FN BMD, whereas TG and FBG were only related to TH BMD (Table 2).

**TABLE 2:** Correlations between clinical factors and BMDs.

Variable	LS BMD		FN BMD		TH BMD	
	*r*	*P*	*r*	*P*	*r*	*P*
Age (years)	–0.230	<0.001	–0.360	<0.001	–0.315	<0.001
Duration (years)	–0.041	0.424	–0.120	0.018	–0.099	0.051
BMI (kg/m^2^)	0.244	<0.001	0.345	<0.001	0.403	<0.001
HbA1c (%)	–0.102	0.047	–0.138	0.007	–0.112	0.028
Creatinine (mg/l)	0.135	0.008	0.036	0.477	0.058	0.250
Cystatin C (mg/L)	–0.033	0.516	–0.083	0.102	–0.107	0.034
Cre/CysC	0.170	0.001	0.178	<0.001	0.205	<0.001
UA (μmol/L)	0.126	0.013	0.140	0.006	0.188	<0.001
TP (g/L)	–0.039	0.441	–0.015	0.776	–0.034	0.508
ALB (g/L)	0.013	0.794	0.051	0.312	0.062	0.225
TBIL (μmol/L)	0.035	0.499	0.085	0.095	0.082	0.109
DBIL (μmol/L)	–0.039	0.447	–0.002	0.974	–0.002	0.966
IDBIL (μmol/L)	0.049	0.335	0.089	0.080	0.087	0.086
TG (mmol/L)	0.059	0.252	0.090	0.076	0.111	0.029
TCH (mmol/L)	0.048	0.353	–0.001	0.987	–0.009	0.858
HDL (mmol/L)	–0.129	0.011	–0.169	0.001	–0.181	<0.001
LDL (mmol/L)	0.050	0.327	0.010	0.841	0.005	0.915
VLDL (mmol/L)	0.077	0.131	0.062	0.228	0.054	0.287
FBG (mmol/L)	–0.031	0.540	–0.076	0.135	–0.105	0.040
FCP (ng/mL)	0.080	0.135	0.072	0.173	0.100	0.060
FT3 (pmol/L)	–0.058	0.264	–0.018	0.731	–0.044	0.392
FT4 (pmol/L)	–0.074	0.150	–0.056	0.274	–0.093	0.068
TSH (mIU/L)	0.035	0.497	0.003	0.959	–0.002	0.975
PTH (pg/mL)	–0.032	0.543	–0.095	0.075	–0.076	0.152
CT (pg/mL)	0.071	0.253	–0.003	0.960	–0.013	0.835
25-(OH)D_3_ (ng/mL)	–0.044	0.397	0.008	0.875	–0.004	0.945
Ca (mmol/L)	–0.021	0.685	–0.048	0.346	–0.031	0.544
P (mmol/L)	0.157	0.002	0.200	<0.001	0.194	<0.001
Mg (mmol/L)	–0.050	0.332	–0.059	0.249	–0.060	0.246

BMI, body mass index; HbA1c, hemoglobin A1c; Cre/CysC, creatinine to cystatin C ratio; UA, uric acid; TP, total protein; ALB, albumin; TBIL, total bilirubin; DBIL, direct bilirubin; IDBIL, indirect bilirubin; TG, triglycerides; TCH, total cholesterol; HDL, high-density lipoproteins; LDL, low-density lipoproteins; VLDL, very low-density lipoprotein; FBG, fasting blood glucose; FCP, fasting C-peptide; FT3, free triiodothyronine; FT4, free thyroxine; TSH, thyroid-stimulating hormone; PTH, parathyroid hormone; CT, calcitonin; 25-(OH)D_3_, 25-hydroxy-vitamin D_3_; Ca, calcium; P, phosphorus; Mg, magnesium; LS BMD, lumbar spine bone mineral density; FN BMD, femoral neck bone mineral density; TH BMD, total hip bone mineral density.

### Multiple stepwise linear regression analyses of variables related to the bone mineral densities

Multiple stepwise linear regression analyses of variables related to the BMDs of LS, FN, and TH are shown in Table 3. We observed that Cre/CysC was the only biochemical predictor of the BMDs of LS (β = 0.137, *p* = 0.01), FN (β = 0.097, *p* = 0.038), and TH (β = 0.145, *p* = 0.002). In addition to Cre/CysC, BMI (LS: β = 0.216, *p* < 0.001; FN: β = 0.278, *p* < 0.001; TH: β = 0.346, *p* < 0.001), age (LS: β = −0.198, *p* < 0.001; FN: β = −0.292, *p* < 0.001; TH: β = −0.264, *p* < 0.001), and sex (LS: β = 0.146, *p* = 0.006; FN: β = 0.296, *p* < 0.001; TH: β = 0.257, *p* < 0.001) were also independent predictors of BMD at the three sites. Moreover, HbA1c (β = −0.094, *p* = 0.026) and serum phosphorus (β = 0.090, *p* = 0.036) were independent predictors of FN BMD. FBG (β = −0.088, *p* = 0.035) was a predictor of TH BMD (Table 3).

**TABLE 3:** Multiple stepwise linear regression analyses of variables related to the BMDs.

Variable	Beta (95% CI)		*P*-value
**LS BMD**			
Age	–0.198	(−0.007, −0.003)	<0.001
BMI	0.216	(0.006, 0.015)	<0.001
Sex	0.146	(0.015, 0.092)	0.006
Cre/CysC	0.137	(0.004, 0.030)	0.010
**FN BMD**			
Age	–0.292	(−0.008, −0.004)	<0.001
Sex	0.296	(0.064, 0.122)	<0.001
BMI	0.278	(0.008, 0.015)	<0.001
HbA1c	–0.094	(−0.013, −0.001)	0.026
P	0.090	(0.005, 0.138)	0.036
Cre/CysC	0.097	(0.001, 0.020)	0.038
**TH BMD**			
BMI	0.346	(0.011, 0.018)	<0.001
Sex	0.257	(0.053, 0.111)	<0.001
Age	–0.264	(−0.007, −0.004)	<0.001
Cre/CysC	0.145	(0.006, 0.025)	0.002
FBG	–0.088	(−0.008, 0.000)	0.035

Adopted factors: sex, age, BMI, HbA1c, Cre/CysC, uric acid, high-density lipoproteins, P for the LS BMD; sex, age, duration, BMI, HbA1c, Cre/CysC, uric acid, high-density lipoproteins, P for the FN BMD; sex, age, BMI, HbA1c, Cre/CysC, uric acid, triglycerides, high-density lipoproteins, FBG, P for the TH BMD. CI, confidence interval; BMI, body mass index; Cre/CysC, creatinine to cystatin C ratio; HbA1c, hemoglobin A1c; P, phosphorus; FBG, fasting blood glucose.

### Subgroup analysis according to sex and bone mass

Considering the significant effect of sex on BMD, Pearson correlation and multivariate regression analysis of BMD were performed according to sex groups (Supplementary Tables 1, 2). The Cre/CysC has a significant correlation with BMD in men (LS: *r* = 0.144, *p* = 0.042; FN: *r* = 0.235, *p* = 0.001; TH: *r* = 0.284, *p* < 0.001), and only significantly correlated with LS BMD (*r* = 0.203, *p* = 0.005) in women; however, the positive correlation trend was observed in the BMDs of FN (*r* = 0.108, *p* = 0.138) and TH (*r* = 0.114, *p* = 0.119) in women. In multiple stepwise linear regression analysis, after adjusting for confounding factors, Cre/CysC was the independent predictor of the BMDs of FN (β = 0.157, *p* = 0.014) and TH (β = 0.21, *p* = 0.001) in men. Meanwhile, it was the independent predictor of LS BMD (β = 0.159, *p* = 0.02) in women.

Patients were further classified based on the *T*-scores as follows: normal, osteopenic, and osteoporotic groups. Of the patients, 140 men (69.3%) and 76 women (40.2%) were diagnosed as normal; 58 men (28.7%) and 81 women (42.9%) were diagnosed with osteopenia; and 4 men (2%) and 32 women (16.9%) were diagnosed with osteoporosis. Older patients, patients with low BMI, and those with low Cre/CysC values were more likely to have osteopenia and osteoporosis. In addition, serum creatinine, UA, ALB, and serum phosphorus levels of patients with osteopenia and osteoporosis were decreased compared to patients with normal *T*-scores. However, the HDL levels of patients with osteopenia and osteoporosis were higher than that of patients with normal *T*-scores. The difference between the groups was statistically significant (Table 4).

**TABLE 4:** Characteristics of patients in the normal, osteopenia, and osteoporosis group.

Variable	Normal (*n* = 216)	Osteopenia (*n* = 139)	Osteoporosis (*n* = 36)	*P*
**SEX**				
MALE	140 (69.3%)	58 (28.7%)	4 (2%)	<0.001
FEMALE	76 (40.2%)	81 (42.9%)	32 (16.9%)	
AGE (years)	58.71 ± 6.38	63.30 ± 7.07	67.69 ± 8.76	<0.001
DURATION (years)	10.50 (5.00, 16.00)	10.00 (6.00, 17.50)	10.00 (2.00, 17.50)	0.856
BMI (kg/m^2^)	26.17 ± 3.73	25.01 ± 3.90	23.44 ± 3.93	<0.001
HBA1C (%)	8.50 ± 1.87	9.26 ± 2.30	9.19 ± 1.89	0.002
CREATININE (mg/L)	7.39 ± 2.67	6.68 ± 2.16	6.19 ± 2.56	0.004
CYSTATIN C (mg/L)	1.09 ± 0.27	1.12 ± 0.29	1.25 ± 0.98	0.077
CRE/CYSC	6.81 ± 1.50	6.01 ± 1.36	5.49 ± 1.40	<0.001
UA (μmol/L)	297.13 ± 97.73	273.15 ± 84.26	235.79 ± 86.53	<0.001
TP (g/L)	65.52 ± 6.41	65.18 ± 6.25	64.46 ± 5.91	0.622
ALB (g/L)	41.94 ± 4.31	41.09 ± 4.52	39.72 ± 4.59	0.011
TBIL (μmol/L)	14.73 ± 5.63	14.25 ± 5.03	12.79 ± 4.38	0.122
DBIL (μmol/L)	3.62 ± 1.65	3.64 ± 1.55	3.76 ± 1.88	0.887
IDBIL (μmol/L)	11.15 ± 4.60	10.64 ± 4.21	9.35 ± 3.17	0.063
TG (mmol/L)	1.28 (0.92, 1.94)	1.19 (0.84, 1.87)	1.17 (0.74, 1.64)	0.363
TCH (mmol/L)	4.28 ± 1.19	4.54 ± 1.30	4.24 ± 1.03	0.123
HDL (mmol/L)	1.21 ± 0.25	1.28 ± 0.29	1.31 ± 0.42	0.017
LDL (mmol/L)	2.56 ± 0.82	2.71 ± 0.87	2.48 ± 0.91	0.153
VLDL (mmol/L)	0.42 (0.32, 0.58)	0.44 (0.31, 0.57)	0.40 (0.30, 0.48)	0.587
FBG (mmol/L)	7.57 ± 3.34	8.33 ± 3.82	7.43 ± 3.22	0.108
FCP (ng/mL)	1.94 ± 1.13	2.00 ± 1.37	1.57 ± 1.17	0.212
FT3 (pmol/L)	4.55 ± 0.74	4.62 ± 2.89	4.22 ± 0.71	0.522
FT4 (pmol/L)	16.79 ± 2.58	17.33 ± 6.39	16.70 ± 2.67	0.490
TSH (miu/L)	2.19 ± 1.74	2.24 ± 1.90	1.95 ± 1.25	0.704
PTH (pg/mL)	34.36 ± 16.37	35.00 ± 15.17	39.65 ± 21.53	0.235
CT (pg/mL)	6.53 (3.75, 12.53)	5.38 (2.86, 11.44)	3.38 (2.16, 5.80)	0.150
25-(OH)D_3_ (ng/mL)	17.65 ± 6.70	17.38 ± 8.32	16.87 ± 6.56	0.826
CA (mmol/L)	2.27 ± 0.12	2.27 ± 0.11	2.26 ± 0.11	0.770
P (mmol/L)	1.26 ± 0.19	1.24 ± 0.22	1.16 ± 0.19	0.020
MG (mmol/L)	0.91 ± 0.08	0.91 ± 0.08	0.94 ± 0.20	0.167
LS BMD (G/CM^2^)	1.17 ± 0.14	0.98 ± 0.14	0.77 ± 0.08	<0.001
FN BMD (G/CM^2^)	0.99 ± 0.12	0.79 ± 0.08	0.68 ± 0.11	<0.001
TH BMD (G/CM^2^)	1.05 ± 0.12	0.84 ± 0.09	0.72 ± 0.12	<0.001

BMI, body mass index; HbA1c, hemoglobin A1c; Cre/CysC, creatinine to cystatin C ratio; UA, uric acid; TP, total protein; ALB, albumin; TBIL, total bilirubin; DBIL, direct bilirubin; IDBIL, indirect bilirubin; TG, triglycerides; TCH, total cholesterol; HDL, high-density lipoproteins; LDL, low-density lipoproteins; VLDL, very low-density lipoprotein; FBG, fasting blood glucose; FCP, fasting C-peptide; FT3, free triiodothyronine; FT4, free thyroxine; TSH, thyroid-stimulating hormone; PTH, parathyroid hormone; CT, calcitonin; 25-(OH)D_3_, 25-hydroxy-vitamin D_3_; Ca, calcium; P, phosphorus; Mg, magnesium; LS BMD, lumbar spine bone mineral density; FN BMD, femoral neck bone mineral density; TH BMD, total hip bone mineral density.

**FIGURE 2:**
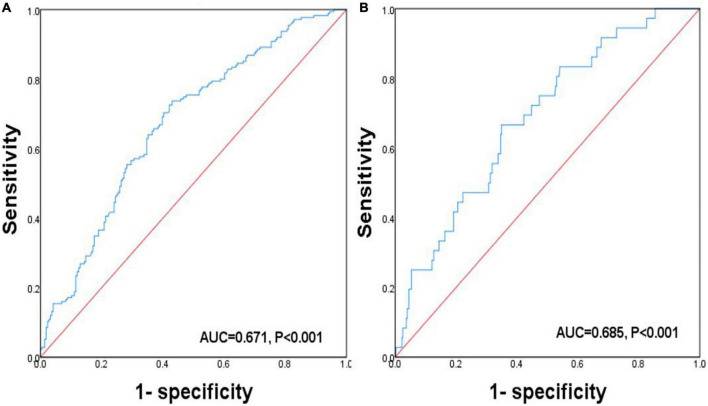
Receiver operating characteristic curve analysis related to the impact of Cre/CysC on the BMDs. **(A)** ROC curve analysis related to the impact of Cre/CysC on the diagnosis of osteopenia. **(B)** ROC curve analysis related to the impact of Cre/CysC on the diagnosis of osteoporosis.

The predictive ability of Cre/CysC about osteopenia and osteoporosis was assessed using ROC curve analysis (Figure 2). Cre/CysC showed moderate strength in predicting both osteopenia and osteoporosis with AUC = 0.671 and 0.685, respectively. The values of Cre/CysC = 6.51 (sensitivity 73.7%, specificity 56.9%) and 5.87 (sensitivity 66.7%, specificity 65.1%) were determined as cut-off points for osteopenia and osteoporosis.

## Discussion

In this cross-sectional study of Chinese patients with T2DM, we found positive correlations between Cre/CysC and LS, FN, and TH BMDs. After adjusting for sex, age, BMI, and other multiple confounding factors such as serum UA and blood phosphorus, it was still an independent predictor of the above indicators. In addition, according to *T*-scores grouping analysis, the Cre/CysC of the osteoporosis group was significantly lower than that of the osteopenia and normal groups. To our knowledge, this is the first time that the correlation between Cre/CysC and BMD has been confirmed in patients with T2DM.

Type 2 diabetes mellitus is an inflammatory chronic condition characterized by insulin resistance and impaired glucose metabolism, as well as complications of multiple organ systems (15). A cohort study reported that the incidence of sarcopenia in patients with T2DM was 15.7%, a significantly higher rate than that in healthy controls (6.9%), and the odds ratio for sarcopenia was 3.06 (16). According to the guideline of European Working Group on Sarcopenia in Older People, sarcopenia can only be diagnosed when a patient has a low muscle strength as well as low muscle mass (5). A major determinant of muscle strength is muscle myosteatosis, which could be reflected by muscle density (high muscle density means low fat infiltration) (17). Therefore, both muscle mass and muscle density are two determining factors for sarcopenia. A previous study on Korean adults with T2DM aged 50 years and older found that the total body muscle mass was an important factor related to FN BMD (2). In patients with T2DM, decreased muscle mass leads to deteriorated insulin sensitivity, aggravated diabetes (18), increased somatostatin secretion, abnormal bone metabolism, and reduced bone mass, and is associated with osteoporosis (19). Moreover, previous studies also reported that compared with the healthy control group, patients with T2DM mainly presented reduced muscle strength and performance, which was related to muscle density (20). The mechanism leading to the decrease of muscle density in patients with T2DM remains unclear, but may be related to insulin resistance and decreased function and number of mitochondria (21, 22).

The circulating Cre/CysC, which can be used as a predictor of myosteatosis and muscle mass (23), was first reported in 2013 and has received widespread attention. It has been increasingly used in the screening of sarcopenia in diabetic and non-diabetic patients. Our previous studies have shown that the Cre/CysC can predict not only the muscle mass but also muscle density in patients with T2DM (24). Based on the close correlation between muscle and bone health, we further confirmed that Cre/CysC could be used as an independent predictor of BMD in the LS, FN, and TH. The positive correlation between Cre/CysC and BMD may be related to the fact that Cre/CysC is an alternative indicator of muscle density and mass; this is consistent with the findings of a small-scale study in patients with primary osteoporosis, which reported that Cre/CysC was positively correlated with LS and FN BMD (3). Furthermore, Komorita et al. confirmed that Cre/CysC was a major risk factor for brittle fractures in patients with T2DM (25). We also observed that the correlation between Cre/CysC and BMD was stronger than that between BMD and creatinine or cystatin C alone, indicating that the Cre/CysC value, rather than serum creatinine or cystatin C level, would be a more appropriate alternative indicator of bone health in patients with T2DM.

**FIGURE 3:**
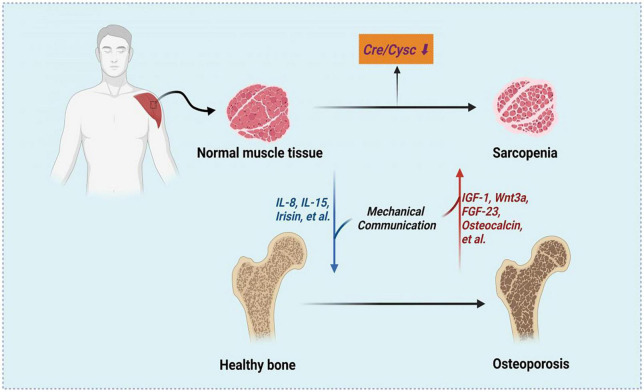
The associations between Cre/CysC, sarcopenia and osteoporosis. Created with BioRender.com.

The relationship between osteoporosis and sarcopenia is reasonable in the context of the bone–muscle subunit and a common mesenchymal precursor of muscle and bone are formed from (26). The crosstalk between muscle and both has been summarized into two major aspects, including mechanical communication and biochemical communication. The mechanical communication has been investigated in detail and plays critical roles during embryonic patterning, postnatal allometric growth, and the homeostatic relationship of adult life and aging (27). Both muscle and bone could be regarded as secretory/endocrine organs. The myokines secreted by muscles may regulate the bone mineral content, such IL-15 (28), IL-8 (29) and irisin (30). On the other side, the factors released by bone including IGF-1 (26), Wnt3a (31), FGF23 (32) and osteocalcin (33), can mediate myogenesis and muscle function. Therefore, as a marker of sarcopenia, Cre/CysC can also predict BMD. The associations and possible mechanisms between Cre/CysC, sarcopenia and osteoporosis were shown in Figure 3.

To further verify the reliability of the conclusion, we conducted a subgroup analysis according to sex and bone mass. When stratified by sex, we found that in men, Cre/CysC was positively correlated with BMD at the three sites and was able to independently predict FN and TH BMD. In women, Cre/CysC was able to independently predict LS BMD and showed tendency for positive correlations with FN and TH BMD. There may be several reasons for the discrepancies in the association between Cre/CysC values and BMD in men and women. First, the number of women enrolled in the study was relatively lower than that of men, and the ability to detect statistical differences was weak. Second, there are significant differences in HbA1c, UA, HDL, and serum phosphorus levels between men and women, which may have weakened the association of Cre/CysC and BMD. Finally, BMI had a positive correlation with all BMDs, whereas age had a negative correlation with all BMDs. In multiple regression analysis, age and BMI as potential confounding factors may weaken the effect of Cre/CysC on BMD. Therefore, the next step is to conduct a large sample study to confirm the effect of Cre/CysC on BMD in different sex.

When stratified by *T*-scores, the Cre/CysC value of the normal bone mass group was the highest, followed by those of the osteopenic and the osteoporotic groups, wherein the difference was significant. The Cre/CysC value decreases with the decrease in bone mass, indicating that Cre/CysC is closely related to BMD. These results are consistent with previous findings that older participants with low BMD levels had increased sarcopenia incidence, decreased muscle strength, low muscle mass, and impaired physical performance (34). Although Cre/CysC showed moderate abilities in predicting osteopenia and osteoporosis in the ROC analysis, it can still help clinicians to avoid unnecessarily DEXA examination because of its low price and convenience of measurement.

In addition to Cre/CysC, this study also found that BMI was positively correlated with BMD, and was an independent predictor for LS, FN, and TH BMDs in patients with T2DM, which is in accordance with previous studies showing that increased BMD in patients with T2DM was caused by increased BMI (35, 36). Patients with T2DM are more prone to obesity and dyslipidemia, and increased BMI may lead to increased bone strain in daily activities. We also noticed a negative correlation between age and BMD, which is reasonable because BMD gradually decreases with increasing age. A significantly higher BMD was observed in men than in women based on the impact of sex differences on BMD. There was a significant positive correlation between serum phosphorus and TH BMD. Previous studies have shown that relatively high blood phosphorus levels in the normal range may be beneficial to BMD (37). Phosphate is important for osteoblast differentiation and extracellular matrix mineralization, with its level directly affecting bone metabolism (38). HbA1c is used as an indicator of diabetes control, which had a significant negative correlation with FN BMD, indicating that patients with poor control of diabetes have lower BMD, higher risk of osteoporosis, and fracture. A cohort study in Taiwan observed similar results as ours, which showed that patients with T2DM and higher HbA1c had a higher risk of fracture (39). FBG is also a common indicator of blood glucose control, and a significant negative correlation was observed with the TH BMD.

In order to eliminate or lessen the interference of sex hormones, our study only included the patients older than 50 and all the women were postmenopausal. In fact, for all women (including those of reproductive age), blood levels of estradiol and testosterone were significant determinants of BMD (40). A decline in estradiol level has been recognized as the most critical hormonal regulator of the menopause-associated decrease in BMD (41). A recent genome-wide study provided further support of the effects of estradiol on BMD in maintaining skeletal health in postmenopausal women (42). The bone-sparing effect of estrogen is antiresorption by inhibition of osteoclast activity, and testosterone, like estrogen, appears to stimulate bone turnover, acting directly or indirectly *via* conversion into estradiol in human osteoblasts to increase androgen receptor expression and stimulate bone cell proliferation and mineralization (43, 44).

This study had a few limitations. First, due to the cross-sectional design, the causal relationship between Cre/CysC and BMDs could not be determined. Therefore, prospective studies are required for further verification. Second, blood biochemical indicators were only measured once at baseline, which may have caused measurement errors. Third, we did not evaluate the muscle mass and function (e.g., handgrip strength and gait speed) and could not further confirm that the ability of Cre/CysC to predict BMD was achieved through the muscle. Finally, this is a single-center study and participants of this study were mainly Chinese Han adults, therefore it is not clear whether our conclusion can be generalized to other ethnic groups.

## Conclusion

In conclusion, the present study firstly demonstrated that the Cre/CysC may be a valuable predictor of BMD in Chinese older adults patients with T2DM. It may help clinicians to avoid unnecessarily DEXA examination and important clinical significance because of its low price and have convenience of measurement.

## Data availability statement

The raw data supporting the conclusions of this article will be made available by the authors, without undue reservation.

## Ethics statement

This study was approved by the Ethics Committee of the Affiliated Hospital of Jining Medical University (2020-BS-008).

## Author contributions

TG and MZ conceived and designed this study. TG collected the data and drafted a manuscript. TG and FL analyzed the data. YH, MJ, and GL contributed to the collection of materials and the revision of the article. FL, QY, BB, and MZ made critical changes to the writing of the article. All authors have access to the database, contributed to this article, and approved its publication.
