# Supplementary material for: Association between the ratio of serum creatinine to cystatin C and bone mineral density in Chinese older adults patients with type 2 diabetes mellitus
Source: Front Nutr. 2022 Oct 21;9:1035853. doi: 10.3389/fnut.2022.1035853 (PMC9634484; doi:10.3389/fnut.2022.1035853)
Supplement: Supplementary file 1 [file Data_Sheet_1.doc]

**Supplemental table 1** Correlations between clinical factors and BMDs stratified by gender

| Variable | **LS BMD** | | **FN BMD** | | **TH BMD** | |
| --- | --- | --- | --- | --- | --- | --- |
|  | r | p | r | p | r | p |
| **Male** |  |  |  |  |  |  |
| Age (years) | -0.162 | 0.022 | -0.268 | <0.001 | -0.238 | 0.001 |
| Duration (years) | -0.038 | 0.595 | -0.096 | 0.176 | -0.089 | 0.208 |
| BMI (kg/m2) | 0.252 | <0.001 | 0.433 | <0.001 | 0.467 | <0.001 |
| HbA1c (%) | -0.101 | 0.159 | -0.174 | 0.014 | -0.162 | 0.022 |
| Creatinine(mg/L) | 0.089 | 0.210 | 0.037 | 0.598 | 0.093 | 0.190 |
| Cystatin C(mg/L) | -0.011 | 0.874 | -0.169 | 0.016 | -0.145 | 0.040 |
| Cre/CysC | 0.144 | 0.042 | 0.235 | 0.001 | 0.284 | <0.001 |
| UA (μmol/L) | 0.052 | 0.461 | 0.149 | 0.034 | 0.213 | 0.002 |
| TP (g/L) | 0.006 | 0.938 | 0.063 | 0.377 | 0.042 | 0.559 |
| ALB (g/L) | 0.031 | 0.663 | 0.109 | 0.125 | 0.092 | 0.197 |
| TBIL (μmol/L) | 0.064 | 0.370 | 0.091 | 0.200 | 0.088 | 0.215 |
| DBIL (μmol/L) | 0.084 | 0.237 | 0.051 | 0.476 | 0.063 | 0.375 |
| IDBIL (μmol/L) | 0.047 | 0.506 | 0.095 | 0.181 | 0.087 | 0.221 |
| TG (mmol/L) | 0.033 | 0.648 | 0.082 | 0.248 | 0.083 | 0.242 |
| TCH (mmol/L) | -0.016 | 0.819 | -0.051 | 0.477 | -0.050 | 0.486 |
| HDL (mmol/L) | -0.164 | 0.021 | -0.222 | 0.002 | -0.234 | 0.001 |
| LDL (mmol/L) | 0.005 | 0.943 | -0.032 | 0.653 | -0.029 | 0.683 |
| VLDL (mmol/L) | 0.038 | 0.597 | 0.051 | 0.479 | 0.048 | 0.503 |
| FBG (mmol/L) | 0.009 | 0.897 | -0.060 | 0.401 | -0.100 | 0.158 |
| FCP (ng/mL) | 0.075 | 0.310 | 0.096 | 0.194 | 0.140 | 0.057 |
| FT3 (pmol/L) | 0.090 | 0.205 | 0.214 | 0.002 | 0.175 | 0.013 |
| FT4 (pmol/L) | 0.086 | 0.226 | 0.094 | 0.189 | 0.053 | 0.461 |
| TSH (mIU/L) | 0.049 | 0.491 | -0.040 | 0.578 | -0.016 | 0.825 |
| PTH (pg/mL) | -0.052 | 0.481 | -0.104 | 0.161 | -0.099 | 0.182 |
| CT (pg/mL) | 0.085 | 0.312 | 0.015 | 0.855 | -0.003 | 0.975 |
| 25-(OH)D3(ng/mL) | -0.028 | 0.700 | -0.063 | 0.381 | -0.081 | 0.260 |
| Ca (mmol/L) | 0.001 | 0.988 | 0.000 | 0.999 | 0.027 | 0.705 |
| P (mmol/L) | 0.089 | 0.213 | 0.165 | 0.021 | 0.119 | 0.097 |
| Mg (mmol/L) | -0.045 | 0.528 | -0.018 | 0.803 | -0.028 | 0.697 |
| **Female** |  |  |  |  |  |  |
| Age (years) | -0.287 | <0.001 | -0.449 | <0.001 | -0.385 | <0.001 |
| Duration (years) | -0.044 | 0.549 | -0.149 | 0.042 | -0.110 | 0.132 |
| BMI (kg/m2) | 0.234 | 0.001 | 0.255 | <0.001 | 0.341 | <0.001 |
| HbA1c (%) | -0.101 | 0.175 | -0.098 | 0.183 | -0.060 | 0.415 |
| Creatinine(mg/L) | 0.179 | 0.014 | 0.035 | 0.634 | 0.023 | 0.756 |
| Cystatin C(mg/L) | -0.046 | 0.535 | -0.039 | 0.590 | -0.093 | 0.205 |
| Cre/CysC | 0.203 | 0.005 | 0.108 | 0.138 | 0.114 | 0.119 |
| UA (μmol/L) | 0.198 | 0.007 | 0.129 | 0.077 | 0.162 | 0.026 |
| TP (g/L) | -0.082 | 0.266 | -0.100 | 0.173 | -0.113 | 0.121 |
| ALB (g/L) | -0.001 | 0.989 | -0.001 | 0.990 | 0.035 | 0.637 |
| TBIL (μmol/L) | -0.001 | 0.987 | 0.077 | 0.293 | 0.073 | 0.319 |
| DBIL (μmol/L) | -0.158 | 0.031 | -0.061 | 0.403 | -0.073 | 0.320 |
| IDBIL (μmol/L) | 0.050 | 0.501 | 0.083 | 0.260 | 0.088 | 0.229 |
| TG (mmol/L) | 0.085 | 0.245 | 0.100 | 0.171 | 0.142 | 0.051 |
| TCH (mmol/L) | 0.111 | 0.130 | 0.058 | 0.432 | 0.036 | 0.625 |
| HDL (mmol/L) | -0.103 | 0.163 | -0.116 | 0.111 | -0.130 | 0.075 |
| LDL (mmol/L) | 0.088 | 0.230 | 0.054 | 0.450 | 0.039 | 0.592 |
| VLDL (mmol/L) | 0.123 | 0.096 | 0.076 | 0.300 | 0.063 | 0.390 |
| FBG (mmol/L) | -0.074 | 0.314 | -0.096 | 0.191 | -0.111 | 0.131 |
| FCP (ng/mL) | 0.077 | 0.319 | 0.048 | 0.527 | 0.060 | 0.431 |
| FT3 (pmol/L) | -0.101 | 0.177 | -0.089 | 0.233 | -0.113 | 0.128 |
| FT4 (pmol/L) | -0.141 | 0.058 | -0.129 | 0.082 | -0.165 | 0.026 |
| TSH (mIU/L) | 0.018 | 0.812 | 0.038 | 0.607 | 0.008 | 0.913 |
| PTH (pg/mL) | -0.018 | 0.820 | -0.085 | 0.266 | -0.055 | 0.473 |
| CT (pg/mL) | 0.077 | 0.411 | -0.016 | 0.868 | -0.008 | 0.928 |
| 25-(OH)D3(ng/mL) | -0.058 | 0.446 | 0.101 | 0.180 | 0.094 | 0.210 |
| Ca (mmol/L) | -0.041 | 0.579 | -0.109 | 0.138 | -0.101 | 0.169 |
| P (mmol/L) | 0.216 | 0.003 | 0.237 | 0.001 | 0.267 | <0.001 |
| Mg(mmol/L) | -0.054 | 0.467 | -0.091 | 0.217 | -0.084 | 0.256 |

BMI, body mass index; HbA1c, hemoglobin A1C; Cre/CysC, creatinine to cystatin C ratio; UA, uric acid;TP,total protein;ALB,albumin;TBIL,total bilirubin;DBIL,direct bilirubin;IDBIL,indirect bilirubin;

TG, triglycerides;TCH, total cholesterol; HDL, high- density lipoproteins;LDL, low- density lipoproteins;VLDL,very low-density lipoprotein;FBG, fasting blood glucose; FCP , fasting C-peptide;

FT3,free triiodothyronine;FT4,free thyroxine;TSH,thyroid-stimulating hormone;PTH, parathyroid hormone;CT,calcitonin;25-(OH)D_3_,25-hydroxy-vitaminD_3_;Ca,calcium;P,phosphorus;Mg,magnesium;

**Supplemental table 2** Multiple stepwise linear regression analyses of variables related to the BMDs stratified by gender.

| Variable | **Beta(95% CI)** | | | **P value** |
| --- | --- | --- | --- | --- |
| **Male** |  |  |  |  |
| LS BMD |  |  |  |  |
| BMI | 0.242 | (0.005,0.016) |  | 0.001 |
| Age | -0.144 | (-0.007,0.000) |  | 0.037 |
| FN BMD |  |  |  |  |
| BMI | 0.402 | (0.011,0.020) |  | <0.001 |
| Age | -0.211 | (-0.007,-0.002) |  | 0.001 |
| Cre/CysC | 0.157 | (0.003,0.028) |  | 0.014 |
| TH BMD |  |  |  |  |
| BMI | 0.433 | (0.012,0.021) |  | <0.001 |
| Cre/CysC | 0.210 | (0.009,0.033) |  | 0.001 |
| Age | -0.170 | (-0.006,-0.001) |  | 0.006 |
| **Female** |  |  |  |  |
| LS BMD |  |  |  |  |
| Age | -0.237 | (-0.009,-0.003) |  | 0.001 |
| BMI | 0.211 | (0.004,0.017) |  | 0.002 |
| Cre/CysC | 0.159 | (0.004,0.045) |  | 0.020 |
| P | 0.151 | (0.014,0.271) |  | 0.030 |
| FN BMD |  |  |  |  |
| Age | -0.410 | (0.010,-0.005) |  | <0.001 |
| BMI | 0.235 | (0.004,0.013) |  | <0.001 |
| P | 0.152 | (0.018,0.197) |  | 0.019 |
| TH BMD |  |  |  |  |
| Age | -0.329 | (0.009,-0.004) |  | <0.001 |
| BMI | 0.314 | (0.007,0.017) |  | <0.001 |
| P | 0.195 | (0.051,0.240) |  | 0.003 |
| FT4 | -0.127 | (0.007,0.000) |  | 0.045 |

Adopted factors:age,BMI,Cre/CysC,high- density lipoproteins for the LS BMD in male; age,BMI,HbA1c,Cre/CysC,uric acid,high- density lipoproteins,free triiodothyronine, P for the FN BMD in male;age,BMI,HbA1c,Cre/CysC,uric acid,high- density lipoproteins, free triiodothyronine for the TH BMD in male;age,BMI,Cre/CysC,uric acid,direct bilirubin,P for the LS BMD in female;age,duration,BMI,P for the FN BMD in female;age,BMI,uric acid,FT4,P for the TH BMD in female.

BMI, body mass index;Cre/CysC, creatinine to cystatin C ratio; P,phosphorus;FT4,free thyroxine.
